# Antimicrobial and Toxic Effects of *Boswellia serrata* Roxb. and *Mentha piperita* Linn. Essential Oils on Vaginal Inhabitants

**DOI:** 10.3390/medicines9120062

**Published:** 2022-12-09

**Authors:** Mirjana A. Bogavac, Tamara M. Perić, Jovana Mišković, Maja Karaman

**Affiliations:** 1 Department of Obstetrics and Gynecology, Clinical Centre of Vojvodina, Faculty of Medicine, University of Novi Sad, Hajduk Veljkova 3, 21000 Novi Sad, Serbia; 2 PHI Hospital “Sveti Vračevi“, Srpske Vojske 53, 76300 Bijeljina, Bosnia and Herzegovina; 3 Faculty of Medicine, University of Novi Sad, Hajduk Veljkova 3, 21000 Novi Sad, Serbia; 4 Department of Biology and Ecology, Faculty of Sciences, University of Novi Sad, Trg Dositeja Obradovića 3, 21000 Novi Sad, Serbia

**Keywords:** antimicrobial activity, *Boswellia*, *Mentha*, essential oils, toxicity, vaginal microbiota

## Abstract

Commercial essential oils (EOs) of incense, *Boswellia serrata* Roxb, and mint, *Mentha piperita* L., were investigated against vaginal bacterial and *Candida albicans* isolates for antimicrobial potential and safety use. The antimicrobial activity of EOs was investigated through a double-dilution micro-plate assay. A brine shrimp assay was used for the determination of toxicity, while the determination of the chemical composition of EOs was carried out using GS–MS. Obtained minimal inhibitory (MIC) and minimal bactericidal concentration (MBC) point to the activity of mint essential oil (EO) against the multi-resistant *P. aeruginosa* isolate (MIC/MBC at 6.25 µL/mL), while MIC and MBC values for other isolates were reached at higher concentrations (25–50 µL/mL). According to the toxicity assay, the incense EO reached the LC_50_ value at 3.07 µL/mL, while mint EO showed higher toxicity at lower concentrations (0.5 µL/mL) and the LC_50_ could not be determined. The highest antimicrobial potential was obtained for incense against *P. aeruginosa.* Although the toxicity assay showed high toxicity of mint EO to the eggs of aquatic crustaceans *Artemia salina*, further testing of EO toxicity is proposed, for example on healthy cell-lines. According to the GC/MS spectrometry, the most represented components of mint EO were the oxygenated hydrocarbons L-menthone (20.86%) and menthol (31.86%), and they could be proposed for further antimicrobial and toxicity investigation.

## 1. Introduction

Vaginal microbiota includes the microorganisms that colonize the vagina, among which the primary colonizing bacteria are of the genus *Lactobacillus*, while the lactic acid they produce protects against infection by pathogenic species [1].

Other bacterial species frequently found in the vagina are the gram-positive cocci: *Atopobium vaginae*, *Peptostreptococcus* spp., *Staphylococcus* spp., *Streptococcus* spp., and *Bacteroides* spp., *Fusobacterium* spp., *Gardnerella vaginalis*, *Prevotella* spp. as well as gram-negative enteric organisms, such as *Escherichia coli* [2]. A healthy vaginal microbiome aids in the prevention of bacterial vaginosis, yeast infections and other possible problems, by maintaining an acidic pH (<4.5), which is unfavorable for the growth of common pathogens, such as *Gardnerella vaginalis*. However, harmful bacteria or an imbalance in bacteria can lead to serious infection. Most women experience a yeast infection sometime during their lifetime, called vaginal candidiasis, which triggers irritation, inflammation, itching, and painful discharge. It is caused by an overgrowth of a commensal yeast *Candida albicans* that naturally lives in the vagina.

Vaginitis and vulvovaginitis are inflammatory conditions caused by bacterial, fungal, or protozoal infections, with various consequences for women’s health. This type of condition, if not properly treated, can lead to serious problems, especially during pregnancy [3]. The therapy of choice usually includes the administration of antibiotics in combination with a vaginal suppository, with the recommendation that in the case of pregnant women, vaginal swabs and clinical markers should be considered before prescribing antibiotics [4]. However, it is a fact that the incidence of vaginal infections has increased during recent years because of the specific sexual behavior which comprises early entry into sexual relationships, as well as frequent changes in sexual partners. Nowadays, one of the most important challenges for human health is the emergence and spread of bacterial resistance to antimicrobial agents [5]. Due to the growth of antibiotic resistance, unconventional antimicrobial agents such as essential oils (EOs) from medicinal plants have become a topic of interest in recent years [6,7,8].

Incense (*Boswellia serrata* Roxb., family: Burseraceae; genus: *Boswellia*), is a deciduous middle-sized tree, which is mostly concentrated in tropical parts of Asia and Africa, with a long history of medicinal and religious uses. In traditional medicine, gummy resin is mentioned as very effective for diarrhea, dysentery, fevers, skin, blood diseases and cardiovascular diseases, mouth sores, bronchitis, asthma, and vaginal discharges [9,10]. Although historically *B. serrata* is recommended for osteoarthritis, juvenile rheumatoid arthritis, soft tissue fibrosis and spondylitis, without any side effects, present knowledge points out many other pharmacological activities, such as anti-inflammatory, anticancer and hypoglycemic activity, and analgesic, and psychopharmacological effects, with nontoxic activity in vivo on monkeys [11]. The gum-resin extracts or essential oil (EO) of the plant (5–10%) could be used as an ingredient in skincare, in perfumery and aromatherapy, but also in treating chronic inflammatory diseases [10,12].

Besides incense, mint (*Mentha piperita* L., family: Lamiaceae; genus: *Mentha*) is also recognized as a valuable herb in traditional therapy. Menthol from mint EO is an ingredient in many cosmetics products, and mint EO (0.3–0.4%) is used in aromatherapy [13,14].

Furthermore, both the increasing use of common antibiotics and the increased resistance of the microorganisms causing infections, lead to the application of alternative therapy [15,16].

The aim of the study was to evaluate the antimicrobial activity and toxicity of two commercial EOs against pathogen isolates of bacteria and yeasts, obtained from women with symptoms of vaginal infections.

## 2. Materials and Methods

### 2.1. Bacterial and Yeasts Strains

Two gram-positive (*S. aureus* ^1^ and *S. aureus* ^2^) and four gram-negative (*E. coli* ^1^ and *E. coli* ^2^, *P. aeruginosa* and *P. mirabilis*) vaginal bacterial isolates, as well as two yeast isolates (*Candida albicans* ^1^ and *Candida albicans* ^2^) were obtained from women with symptoms of vaginal infection. All isolates were sampled at regular gynecological examinations at the Department of Obstetrics and Gynecology, the Clinical Centre of Vojvodina, Faculty of Medicine, University of Novi Sad, approved by the Ethical Board of the Clinical Centre of Vojvodina.

### 2.2. Essential Oils

*B. serrata* Roxb.—incense and *M. piperita* L.—mint EOs were commercially purchased from the Manufacturer Probotanik, Belgrade, Serbia Seri. No. KE-0713 [17].

### 2.3. Antimicrobial Assay

Antimicrobial activity was determined by the standard microdilution CLSI procedure [18]. For the assay in a 96-well micro-plate (Spektar, Čačak, Serbia), Müller Hinton broth for bacterial (MHB, Torlak, Beograd, Serbia) and malt broth for yeasts (MB, Torlak, Beograd, Serbia) were used. Inoculum was prepared from overnight cultures in accordance with the McFarland standard procedures (1.5 × 10^8^ CFU/mL for bacteria and 1.5 × 10^6^ for yeast). Double dilutions of EOs were prepared in 1% polysorbate (Tween 80) to a final concentration ranging from 1.25 to 50 µL/mL. After incubation at 37 °C/24 h for bacterial and 37 °C/48 h for the yeasts, the number of viable microorganisms was determined. Minimal inhibitory (MIC) and minimal bactericidal/fungal concentration (MBC/MFC) were determined for the EOs, as well as for four commonly used antibiotics (streptomycin, ampicillin, tetracycline and cefuroxime, Torlak, Belgrade, Serbia) and one antimycotic (Nystatin, Hemofarm, Vršac, Serbia) according to standard procedure CLSI [19]. Antibiotics were tested at the final concentration (64–512 µL/mL), while for nystatin the concentration range was from 32 to 256 µL/mL.

### 2.4. Brine-Shrimp Toxicity Assay

The brine-shrimp toxicity bioassay was performed in accordance with Meyer et al. [20]. The investigation of toxicity of EOs was performed in a 96-well micro-plate (Spektar, Čačak). A volume of 25 µL of EO (diluted in 1% dimethyl sulfoxide, ranging from 3.12–50 µL/mL) and 225 µL of artificial sea water (ASW) with approximately ten larvae of brine shrimp *Artemia salina* were added to each well of the micro-plate. The assay was performed in five repetitions for every investigated concentration, with 250 µL ASW with larvae, without EOs as control. After incubation at 30 °C, under constant illumination and aeration the toxicity was calculated by counting the number of of dead nauplii after 24 h, and after adding 50 µL of absolute methanol as control tests, counting all the nauplii present in each well (Stereomicroscope, Zeiss, Germany). Results were expressed as LC_50_ concentration, in accordance with the following Abbott’s equation:

Mortality (%) = ((n test − n control)/n control) × 100

where n test represents the numbers of dead larvae in the first test counting;

n control represents the numbers of all dead larvae after adding methanol (control tests).

### 2.5. Gas Chromatography–Mass Spectrometry (GC/MS)

Analyses of EOs include the preparation of 1:10 *w/v* dilution in dichloromethane and were performed on an Agilent capillary gas chromatograph, coupled with a mass spectrometer (MSD) (model GC Agilent 7890A; MS 5975C, Agilent, Santa Clara, CA, USA). A non-polar fused-silica capillary column (DB-5MS, 5% Phenyl Methyl Siloxan, 60 m × 250 µm × 0.25 µm) was used, under the condition of the volume of injected specimen 1 μL, flow rate 0.81185 mL/min (constant flow mode) with He (carrier gas) in split ratio 50:1. The temperature-program ramp was from 70 °C (2 min) to 150 °C, with a gradient of 10 °C/min and up to 280 °C. The final temperature was kept for 39.33 min and ionization energy was 70 eV in the EI (electron impact) ionization mode, and ion-source temperature 230 °C, scan mass-range of *m*/*z* 30–550 with detector temperature 280 °C. Retention indexes (RI) were used for identification of the EOs components by comparing obtained spectral data of EOs with the RI of the literature data. The laboratory database and mass spectra generated by the instrument software—NIST mass spectroscopy and MSD Chem Station E.02.00.493—were used. Identified components were additionally confirmed using the AMDIS software program (version 2.66 NIST, Gaithersburg, MD, USA) and we verified the mass spectra of the EOs using the Wiley Registry of Mass Spectral Data 7th edition, NIST08 Mass Spectral Library v.2.0f Chemdata, Nist, Gaithersburg, MD, USA.

### 2.6. Statistical Analyses

One-way ANOVA with post-hoc Tukey HSD test at the level of significance *p* < 0.01 was used to determine significant differences between the obtained results of the antimicrobial testing. LC_50_ values for the brine-shrimp bioassay were obtained from linear regression analysis (OriginLab version 8.0, Corporation, Northampton, MA, USA).

## 3. Results

### 3.1. Antimicrobial Activity of Investigated EOs

The estimation of antimicrobial activity generally showed that mint EO proved to be a better antimicrobial agent than incense EO (Table 1). Lower MIC or MBC/MFC values point to the stronger effect of investigated EO against certain bacteria/yeast isolates. MIC and MBC values for mint oil ranged from 6.25 to 50 μL/mL, while incense EO showed only MIC values (at 50 μL/mL) with MBC estimated above this concentration. MIC and MFC activity of EOs point out the strain specificities of *C. albicans* isolates (Table 1). More precisely, the *C. albicans* ^1^ isolate was more resistant to the investigated EOs than the *C. albicans* ^2^. Regarding antibiotic susceptibility, the tested gram-negative strains *E. coli* ^1^, *E. coli* ^2^ and *P. aeruginosa* proved to be the most resistant to all tested antibiotics (Table 1). Nystatin, the only tested antimycotic, exhibited better antifungal activity against *C. albicans* ^1^ than the *C. albicans* ^2^ isolate, the opposite to the tested EOs (Table 1). *C. albicans* ^2^ showed the best susceptibility towards both Eos, and could be suitable for further application in possible alternative therapies.

### 3.2. Brine-Shrimp Toxicity Assay

**Figure 1:**
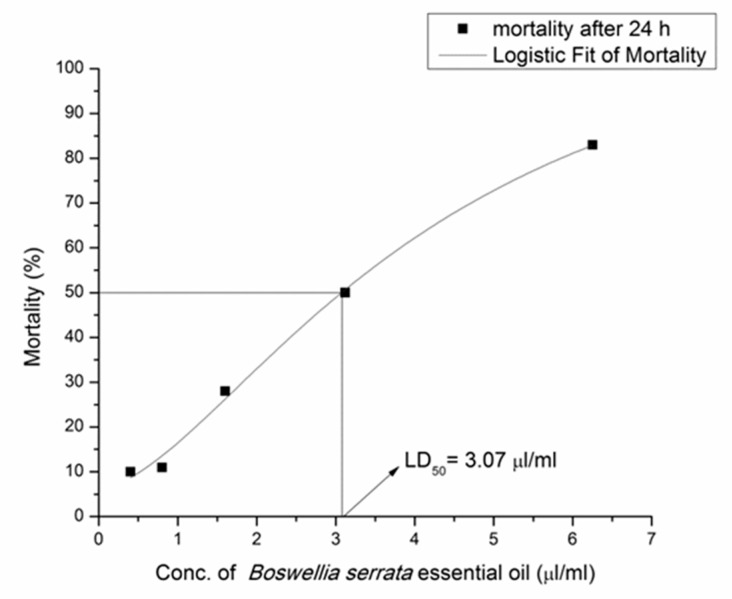
Relationship between mortality (%) and *B. serrata* EO concentration (µL/mL) after 24 h. Mortality after 24 h (■), logistic Fit of Mortality (–––) and LD_50_ = 3.07 µL/mL.

**Figure 2:**
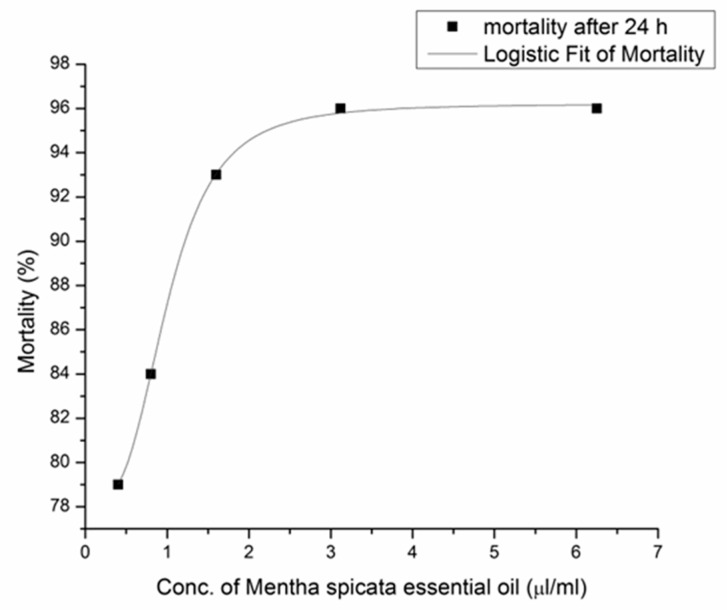
Relationship between mortality (%) and *M. piperita* EO concentration (µL/mL) after 24 h. Mortality after 24 h (■) and logistic Fit of Mortality (–––).

Obtained results for the toxicity assay (LC_50_ values) were based on the relationship between mortality (%) and oil concentration (0.40–6.25 µL/mL) after 24 h. The EO of *B. serrata* reached LC_50_ at 3.07 µL/mL (Figure 1), while *M. piperita* EO exhibited very high toxicity on *A. salina* larvae, even at the lowest investigated concentration (0.40 µL/mL) (Figure 2).

### 3.3. Chemical Composition of EOs

The chemical profile of investigated EOs is presented in Table 2. For EO of incense (*B. serrata*), 24 compounds were identified, representing 89.89% of the total oil composition, while for mint (*M. piperita*), 25 components (92.77%) were identified. *B. serrata* EO contains 52.05% monoterpene hydrocarbons, 19.90% cyclic terpenes and 7.29% of oxygenated hydrocarbons. The main constituents are *β*-thujene (29.77%) and its structural isomer α-thujene (11.71%), followed by monoterpene hydrocarbon α-pinene (8.44%). *M. piperita* EO contains 78.92% of oxygenated hydrocarbons, 6.26% of terpenoids and sesquiterpenes and 3.15% of monoterpene hydrocarbons.

## 4. Discussion

In contrast to the results of antibacterial activity of EO from the bark of *B. serrata* that exhibited significant inhibitory activity against *S. aureus, E. coli* and *P. mirabilis* at 5 µL/mL [21], in the present study incense EO reached MIC only at the highest investigated concentration (50 μL/mL,) and MBC above this concentration. On the contrary, mint EO showed very high activity against *P. aeruginosa* at 6.25 μL/mL, while in other research from India [22] MIC and MBC values were significantly higher (from 200 to 400 µL/mL). 

*Candida* strains showed sensitivity to mint and incense EOs in a range from 12.50 µL/mL to 100 µL/mL (MIC and MFC values), while in another study from Iran, mint EO exhibited antifungal activity against *C. albicans* at much lower MIC/MFC concentrations, at 1.5 µL/mL and 3.3 µL/mL, respectively [23].

In accordance with the antibiotic resistance of clinical isolates, gram-negative bacteria *P. aeruginosa*, *E. coli* ^1^ and *E. coli* ^2^ showed multi resistance to all tested antibiotics, which points to the high resistance of clinical isolates to conventional antibiotics. The *P. aeruginosa* strain showed high susceptibility to the mint EO (6.25 μL/mL), and for that reason the possible alternative use of this EO in the prevention of infection caused by this vaginal bacterium could be recommended.

The number of identified components in EOs varies widely, and may be attributed to many factors, e.g., different genotype, ecotype, temperature, humidity, photoperiod or harvesting time [8]. In the comparative study of commercial EO *B. serrata* with the wild-habitat EO, it is pointed out that the commercial samples contained a higher percentage of monoterpene hydrocarbons (81.90–88.10%), including α-thujene (61.4–69.8%) as the major compound, while the wild-habitat EO contained more oxygenated monoterpenoids/benzenoids (15.70%) and sesquiterpenes (19.20%) [24,25]. In this research, the monoterpene group was dominated by *B. serrata* EO, which is in accordance with the previous study, although some differences in the content of the major constituents exist, such as the highest amount of *β*-Thujene (29.77) and α-Thujene (11.71) in this study. The EO of *B. serrata* predominantly comprised monoterpenoids, of which α-pinene (73.3%) was the major constituent, while other monoterpenoids identified included *β*-pinene (2.05%), cis-verbenol (1.97%), trans-pinocarveol (1.80%), borneol (1.78%), myrcene (1.71%), verbenone (1.71%), limonene (1.42%), thuja-2,4(10)-diene (1.18%) and p-cymene (1.0%), while -copaene (0.13%) was the only sesquiterpene identified in the oil [11].

It has already been observed that there is a large variation in the chemical composition of oils because of different methods of extraction or packaging and storage processes of EOs [8], and thus a series of chemo types have been described. Menthone (32.43%) and 1,8-cineole (18.79%) were the major constituents of the *M. piperita* EO which originated in Egypt [26], while for the same species originating in Serbia and Iran, menthol was the main component, reaching 37.4% and 53.28%, respectively [23,27]. This result from the literature data is in accordance with this study (31.86%), since the major constituents of mint EO were menthol (31.86%), and L-menthone (20.86%), followed by eucalyptol (5.48%) and caryophyllene (3.39%).

Regarding the antimicrobial activity of detected active-compounds, it was found that monoterpene menthol is able to interact with phospholipidic membranes of bacteria, causing disruption of the cytoplasmic membrane and thereby achieving its antimicrobial activity [28]. In the same investigation, menthol showed antimicrobial effect against both gram-positive (*S. aureus*) and gram-negative bacteria *E. coli* [28]. Although menthol as an individual compound is very important in the expression of the antimicrobial activity observed, it is important to underline the fact that synergistic effects of minor components also play an important role [29].

In accordance with the fact that isolated pathogens are susceptible to investigated EOs, unlike certain antibiotics herein tested, they may represent a promising alternative treatment for vaginal infections. Both EOs tested, and especially *M. piperita* EO, expressed promising activity against multi-resistant *P. aeruginosa*, *E. coli* ^1^ and *E. coli* ^2^. Although a number of trials is needed for their acceptance in general clinical practice, *B. serrata* EOs represent a very important alternative source of antimicrobial substances. However, according to the *A. salina* assay that was used in the evaluation of EO acute-toxicity, *B. serrata* EO showed LC_50_ at 3.07 µL/mL and *M. piperita* EO showed an even higher toxicity with LC_50_ at approximately the lowest concentration applied. Hence, further toxicology trials are needed for predicting local vaginal acute-toxicity, using cell lines or in vivo testing.

We should emphasize that many external factors affect the chemical composition of EOs, including different manufacturers’ procedures, the geographical origin of the plant, environmental conditions (mostly climate factors), the point of harvest or other processing-dependent influences, and the extraction type, etc. [8]. Furthermore, we assume that the real effect might be significantly weaker compared to isolated active-compounds, but pure compounds are not generally commercially available. On the contrary, EOs are more commercially accessible, and they represent the mixture of active constituents whose synergistic effect of minor and major constituents show high activity and can be used in the form of additive constituents of vaginalettes. These findings enhance the need for chemical characterizations of EOs to identify the active compounds and their interdependencies, making the optimization and standardization regarding the antimicrobial activity of EO imminent for application. On the other hand, the natural origin makes EO an attractive alternative for the treatment of vaginal infections, and should draw more attention in the future.

## 5. Conclusions

This study provides insight into the antimicrobial activity, as well as toxicity, of *B. serrata* and *M. piperita* commercial EO against vaginal pathogens, with the aim of combatting the phenomenon of antibiotic resistance by promoting the use of alternative treatments in gynecology. Obtained results suggest stronger antibacterial activity of tested EOs compared to tested antibiotics, while the antifungal activity of EOs point to the strain specificities of tested *C. albicans* isolates. Furthermore, comparing the activity of tested EOs, mint stood out as a stronger antibacterial agent, especially as it has shown favorable activity against multi resistant *P. aeruginosa*. This activity may be related to the major constituents of mint EO (menthol and L-menthone), but results in this study strongly suggest that the synergistic effect of minor components also plays an important role. Since *M. piperita* EO exhibited high toxicity on *A. salina* larvae, further cytotoxic-model-system investigations on healthy cell-lines should be performed, to check the real in vivo toxicity. Due to the susceptibility of isolated pathogens to the investigated EOs, both *B. serrata* and *M. piperita* EO may represent an alternative therapeutic agent for the treatment of vaginal infections in the form of future constituents of vaginalettes, but future in vivo trials are needed.

## Figures and Tables

**Table 1:** MIC and MBC/MFC values (µL/mL) of EOs against bacterial and *Candida* strains.

	*B. serrata* Roxb.		*M. piperita* L.		Str		Amp		Tet		Cef		Nyst	
Bacterial Strains *	MIC	MBC	MIC	MBC	MIC	MBC	MIC	MBC	MIC	MBC	MIC	MBC		
	(µL/mL)								(µL/mL)					
*E. coli* ^1^	50 ^a^	↑50 ^b^	50 ^a^	↑50 ^b^	nd	nd	nd	nd	nd	nd	nd	nd		
*E. coli* ^2^	50 ^a^	↑50 ^b^	50 ^a^	50 ^a^	nd	nd	nd	nd	nd	nd	512 ^c^			
*S. aureus* ^1^	50 ^b^	↑50 ^c^	25 ^a^	50 ^b^	128 ^d^	256 ^e^	128 ^d^	128 ^d^	64 ^b^	128 ^d^	512 ^f^			
*S. aureus* ^2^	50 ^b^	↑50 ^c^	25 ^a^	25 ^a^	128 ^d^	256 ^e^	nd	nd	128 ^d^	256 ^e^	nd	nd		
*P. aeruginosa*	50 ^b^	↑50 ^c^	6.25 ^a^	6.25 ^a^	nd	nd	nd	nd	nd	nd	nd	nd		
*P. mirabilis*	50 ^b^	↑50 ^c^	25 ^a^	25 ^a^	128 ^d^	256 ^e^	128 ^d^	256 ^e^	128 ^d^	256 ^d^	512 ^e^	nd		
Fungal Strains *	**MIC**	**MFC**	**MIC**	**MFC**									**MIC**	**MFC**
	(µL/mL)												(µL/mL)	
*C. albicans* ^1^	100 ^b^	↑100 ^c^	100 ^b^	100 ^b^									64 ^a^	64 ^a^
*C. albicans* ^2^	12.50 ^a^	12.50 ^a^	12.50 ^a^	12.50 ^a^									64 ^b^	128 ^c^

* strains-isolates; nd—not detected, meaning resistance R in the tested range of concentration (1.25–50 µL/mL); ↑—higher than the highest value applied MIC—minimum inhibitory concentration; MBC—minimum bactericidal concentration; MFC—minimum fungicidal concentration; Str—streptomycin; Amp—ampicillin; Tet—tetracycline; Cef—cefuroxime, Nyst—nystatin; ^a, b, c, d, e, f^ —letters means significant differences between antimicrobial activity within strain of bacteria determined by Tukey HSD test, at *p* < 0.01. In each row different letters mean significant differences.

**Table 2:** Chemical composition of EOa of *B. serrata* and *M. piperita*.

Peak No.	Compound	R.I. ^a^	EOs (%)	
			*B. serrata*	*M. piperita*
	**Monoterpene hydrocarbons**		**52.05**	**3.15**
1	*β*-Thujene	8.11	**29.77**	-
2	α-Thujene	8.15	**11.71**	-
3	α-Pinene	8.29	8.44	0.44
4	Thuja-2,4(10)-diene	8.40	0.34	-
5	*β*-Myrcene	8.97	1.01	0.10
6	*β*-Pinene	9.03	-	0.82
7	cis-Ocimene	9.46	-	-
8	γ-Terpinene	10.24	-	0.11
9	Dehydro sabinene ketone	11.49	0.78	-
10	Pulegone	13.85	-	1.48
11	Dihydro-4-Carane	14.32	-	0.20
	**Aromatic monoterpenes**		**8.74**	**0.88**
12	o-Cymene	9.59	0.24	
13	m-Cymene	9.69		0.88
14	p-Cymene	9.70	8.50	
	**Cyclic terpenes**		**19.90**	**2.82**
15	Camphene	8.59	0.16	-
16	*β*-Phellandrene	8.86	-	0.20
17	Sabinene	8.87	8.89	-
18	laevo-*β*-Pinene	9.03	0.66	-
19	α-Phellandrene	9.41	0.13	-
20	δ-3-Caren	9.46	5.76	-
21	dl-Limonene	9.78	4.30	2.62
22	trans-Pinocarveol	12.00	-	-
	**Oxygenated hydrocarbons**		**7.29**	**78.92**
23	3-methyl-cyclohexanol	8.44	-	1.18
24	Eucalyptol	9.93	-	5.48
25	*β*-Linalool	10.84	-	-
26	α-Thujone	11.43	0.78	-
27	L-Menthone	12.26	-	**20.86**
28	(-)-cis-Sabinol	12.37	0.20	-
29	dl-Menthone	12.43	-	10.14
30	Menthol	12.66		**31.86**
31	Terpinen-4-ol	12.67	2.05	-
32	p-Menth-1-en-4-ol	12.68	-	-
33	(+)-Isomenthol	12.89	-	1.71
34	p-Menth-1-en-8-ol	12.91	-	-
35	p-Allylanisole	12.92	3.83	-
36	m-Cumenol	13.28	0.15	-
37	Menthyl acetate	14.67	-	7.51
38	Isomenthyl acetate	15.07		0.18
39	O-Methyleugenol	16.72	0.28	
	**Terpenoids and sesquiterpenes**		**1.91**	**6.26**
40	3,9-Epoxy-p-mentha-3,8-diene	12.36	-	1.93
41	S-Carvone	13.93	-	0.19
42	3-carvomenthenone	14.17	-	0.25
43	α-Copaene	16.62	0.21	0.14
44	*β*-Bourbonene	16.84	1.38	-
45	γ-Caryophyllene	17.25	-	0.18
46	trans-α-Bergamotene	17.57	0.18	--
47	*β*-caryphyllene	17.59	-	3.39
48	α-Caryophyllene	18.24	-	0.18
49	α-Amorphene	18.45	0.14	-
	**Oxygenated sesquiterpenes**		-	**0.74**
50	Caryophyllene oxide	20.49	-	0.74
	**Amount of identified compounds**		**89.89**	92.77

^a^ Retention indexes relative to C_9_–C_24_ n-alkanes on the HP 5MS column.

## Data Availability

Not applicable.
